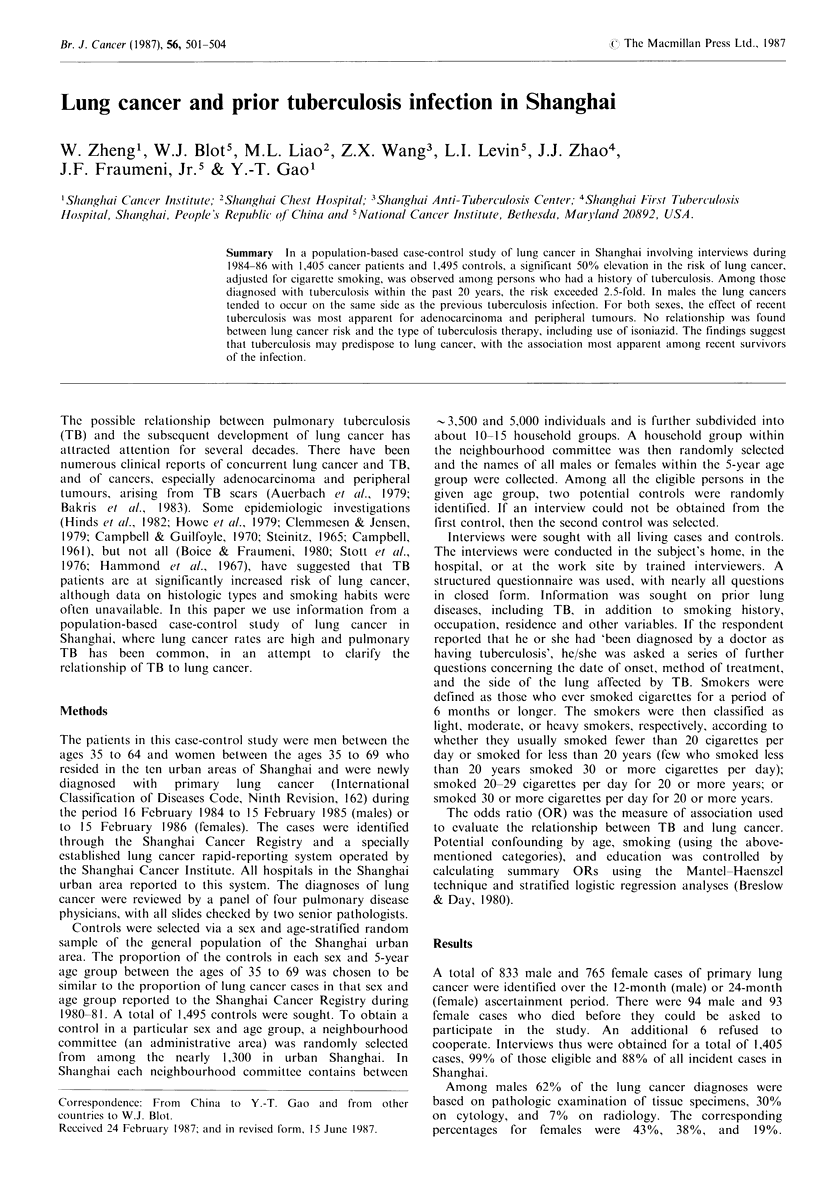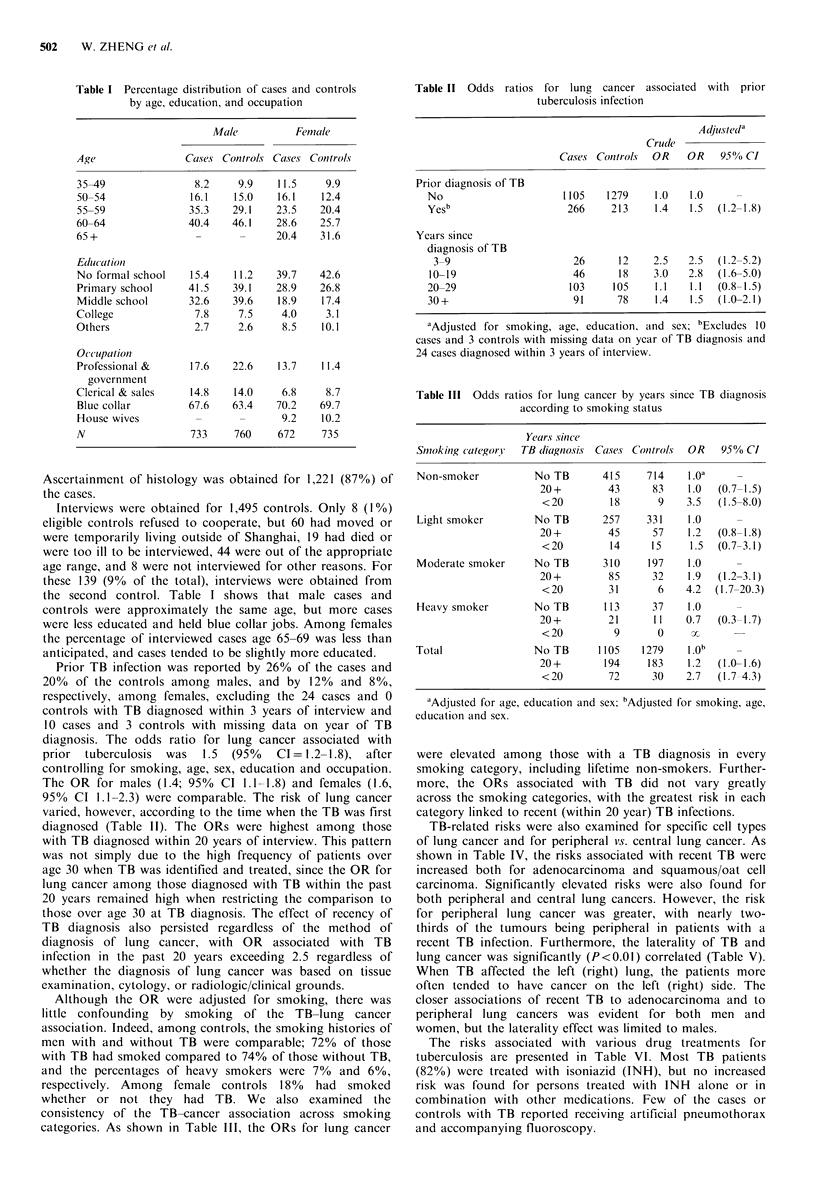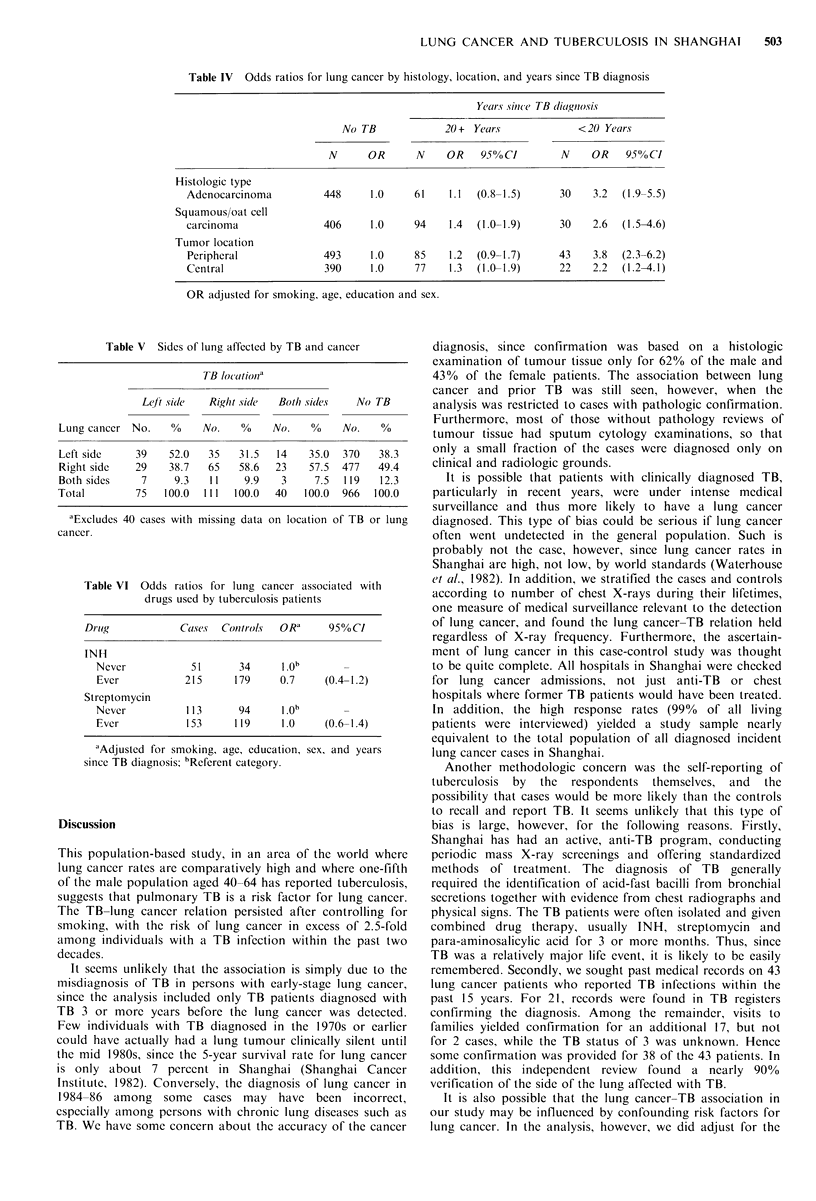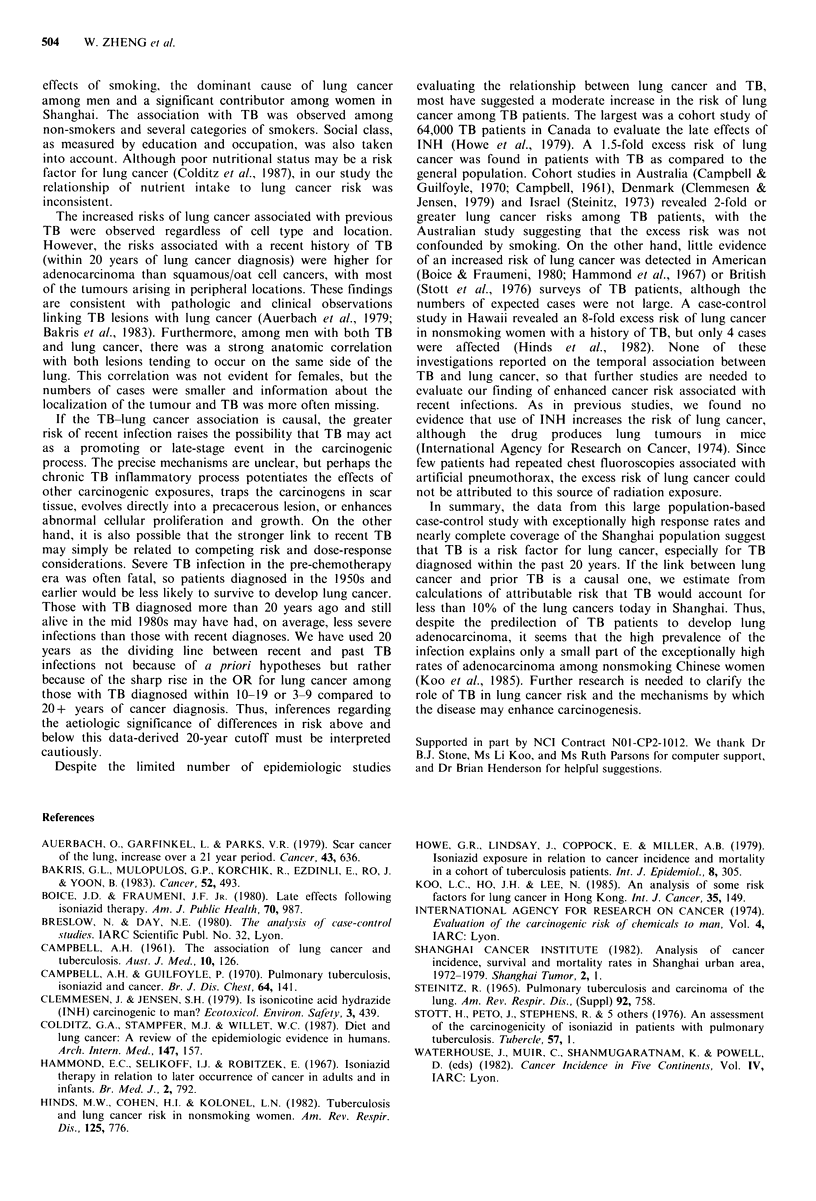# Lung cancer and prior tuberculosis infection in Shanghai.

**DOI:** 10.1038/bjc.1987.233

**Published:** 1987-10

**Authors:** W. Zheng, W. J. Blot, M. L. Liao, Z. X. Wang, L. I. Levin, J. J. Zhao, J. F. Fraumeni, Y. T. Gao

**Affiliations:** Shanghai Cancer Institute, People's Republic of China.

## Abstract

In a population-based case-control study of lung cancer in Shanghai involving interviews during 1984-86 with 1,405 cancer patients and 1,495 controls, a significant 50% elevation in the risk of lung cancer, adjusted for cigarette smoking, was observed among persons who had a history of tuberculosis. Among those diagnosed with tuberculosis within the past 20 years, the risk exceeded 2.5-fold. In males the lung cancers tended to occur on the same side as the previous tuberculosis infection. For both sexes, the effect of recent tuberculosis was most apparent for adenocarcinoma and peripheral tumours. No relationship was found between lung cancer risk and the type of tuberculosis therapy, including use of isoniazid. The findings suggest that tuberculosis may predispose to lung cancer, with the association most apparent among recent survivors of the infection.


Br. J. Cancer (1987), **56**, 501–504 © The Macmillan Press Ltd., 1987

### Lung cancer and prior tuberculosis infection in Shanghai

W. Zheng[1], W.J. Blot[5], M.L. Liao[2], Z.X. Wang[3], L.I. Levin[5], J.J. Zhao[4], J.F. Fraumeni, Jr.[5] & Y.-T. Gao[1]

[1]*Shanghai Cancer Institute;* [2]*Shanghai Chest Hospital;* [3]*Shanghai Anti-Tuberculosis Center;* [4]*Shanghai First Tuberculosis Hospital, Shanghai, People's Republic of China and* [5]*National Cancer Institute, Bethesda, Maryland 20892, USA.*

**Summary** In a population-based case-control study of lung cancer in Shanghai involving interviews during 1984–86 with 1,405 cancer patients and 1,495 controls, a significant 50% elevation in the risk of lung cancer, adjusted for cigarette smoking, was observed among persons who had a history of tuberculosis. Among those diagnosed with tuberculosis within the past 20 years, the risk exceeded 2.5-fold. In males the lung cancers tended to occur on the same side as the previous tuberculosis infection. For both sexes, the effect of recent tuberculosis was most apparent for adenocarcinoma and peripheral tumours. No relationship was found between lung cancer risk and the type of tuberculosis therapy, including use of isoniazid. The findings suggest that tuberculosis may predispose to lung cancer, with the association most apparent among recent survivors of the infection.

The possible relationship between pulmonary tuberculosis (TB) and the subsequent development of lung cancer has attracted attention for several decades. There have been numerous clinical reports of concurrent lung cancer and TB, and of cancers, especially adenocarcinoma and peripheral tumours, arising from TB scars (Auerbach *et al.*, 1979; Bakris *et al.*, 1983). Some epidemiologic investigations (Hinds *et al.*, 1982; Howe *et al.*, 1979; Clemmesen & Jensen, 1979; Campbell & Guilfoyle, 1970; Steinitz, 1965; Campbell, 1961), but not all (Boice & Fraumeni, 1980; Stott *et al.*, 1976; Hammond *et al.*, 1967), have suggested that TB patients are at significantly increased risk of lung cancer, although data on histologic types and smoking habits were often unavailable. In this paper we use information from a population-based case-control study of lung cancer in Shanghai, where lung cancer rates are high and pulmonary TB has been common, in an attempt to clarify the relationship of TB to lung cancer.

### Methods

The patients in this case-control study were men between the ages 35 to 64 and women between the ages 35 to 69 who resided in the ten urban areas of Shanghai and were newly diagnosed with primary lung cancer (International Classification of Diseases Code, Ninth Revision, 162) during the period 16 February 1984 to 15 February 1985 (males) or to 15 February 1986 (females). The cases were identified through the Shanghai Cancer Registry and a specially established lung cancer rapid-reporting system operated by the Shanghai Cancer Institute. All hospitals in the Shanghai urban area reported to this system. The diagnoses of lung cancer were reviewed by a panel of four pulmonary disease physicians, with all slides checked by two senior pathologists.

Controls were selected via a sex and age-stratified random sample of the general population of the Shanghai urban area. The proportion of the controls in each sex and 5-year age group between the ages of 35 to 69 was chosen to be similar to the proportion of lung cancer cases in that sex and age group reported to the Shanghai Cancer Registry during 1980–81. A total of 1,495 controls were sought. To obtain a control in a particular sex and age group, a neighbourhood committee (an administrative area) was randomly selected from among the nearly 1,300 in urban Shanghai. In Shanghai each neighbourhood committee contains between ~3,500 and 5,000 individuals and is further subdivided into about 10–15 household groups. A household group within the neighbourhood committee was then randomly selected and the names of all males or females within the 5-year age group were collected. Among all the eligible persons in the given age group, two potential controls were randomly identified. If an interview could not be obtained from the first control, then the second control was selected.

Interviews were sought with all living cases and controls. The interviews were conducted in the subject's home, in the hospital, or at the work site by trained interviewers. A structured questionnaire was used, with nearly all questions in closed form. Information was sought on prior lung diseases, including TB, in addition to smoking history, occupation, residence and other variables. If the respondent reported that he or she had 'been diagnosed by a doctor as having tuberculosis', he/she was asked a series of further questions concerning the date of onset, method of treatment, and the side of the lung affected by TB. Smokers were defined as those who ever smoked cigarettes for a period of 6 months or longer. The smokers were then classified as light, moderate, or heavy smokers, respectively, according to whether they usually smoked fewer than 20 cigarettes per day or smoked for less than 20 years (few who smoked less than 20 years smoked 30 or more cigarettes per day); smoked 20–29 cigarettes per day for 20 or more years; or smoked 30 or more cigarettes per day for 20 or more years.

The odds ratio (OR) was the measure of association used to evaluate the relationship between TB and lung cancer. Potential confounding by age, smoking (using the above-mentioned categories), and education was controlled by calculating summary ORs using the Mantel–Haenszel technique and stratified logistic regression analyses (Breslow & Day, 1980).

### Results

A total of 833 male and 765 female cases of primary lung cancer were identified over the 12-month (male) or 24-month (female) ascertainment period. There were 94 male and 93 female cases who died before they could be asked to participate in the study. An additional 6 refused to cooperate. Interviews thus were obtained for a total of 1,405 cases, 99% of those eligible and 88% of all incident cases in Shanghai.

Among males 62% of the lung cancer diagnoses were based on pathologic examination of tissue specimens, 30% on cytology, and 7% on radiology. The corresponding percentages for females were 43%, 38%, and 19%.

Correspondence: From China to Y.-T. Gao and from other countries to W.J. Blot.
Received 24 February 1987; and in revised form, 15 June 1987.

**Table I** Percentage distribution of cases and controls by age, education, and occupation

| | *Male* | | *Female* | |
|---|---|---|---|---|
| *Age* | *Cases* | *Controls* | *Cases* | *Controls* |
| 35–49 | 8.2 | 9.9 | 11.5 | 9.9 |
| 50–54 | 16.1 | 15.0 | 16.1 | 12.4 |
| 55–59 | 35.3 | 29.1 | 23.5 | 20.4 |
| 60–64 | 40.4 | 46.1 | 28.6 | 25.7 |
| 65+ | – | – | 20.4 | 31.6 |
| *Education* | | | | |
| No formal school | 15.4 | 11.2 | 39.7 | 42.6 |
| Primary school | 41.5 | 39.1 | 28.9 | 26.8 |
| Middle school | 32.6 | 39.6 | 18.9 | 17.4 |
| College | 7.8 | 7.5 | 4.0 | 3.1 |
| Others | 2.7 | 2.6 | 8.5 | 10.1 |
| *Occupation* | | | | |
| Professional & government | 17.6 | 22.6 | 13.7 | 11.4 |
| Clerical & sales | 14.8 | 14.0 | 6.8 | 8.7 |
| Blue collar | 67.6 | 63.4 | 70.2 | 69.7 |
| House wives | – | – | 9.2 | 10.2 |
| *N* | 733 | 760 | 672 | 735 |

Ascertainment of histology was obtained for 1,221 (87%) of the cases.

Interviews were obtained for 1,495 controls. Only 8 (1%) eligible controls refused to cooperate, but 60 had moved or were temporarily living outside of Shanghai, 19 had died or were too ill to be interviewed, 44 were out of the appropriate age range, and 8 were not interviewed for other reasons. For these 139 (9% of the total), interviews were obtained from the second control. Table I shows that male cases and controls were approximately the same age, but more cases were less educated and held blue collar jobs. Among females the percentage of interviewed cases age 65–69 was less than anticipated, and cases tended to be slightly more educated.

Prior TB infection was reported by 26% of the cases and 20% of the controls among males, and by 12% and 8%, respectively, among females, excluding the 24 cases and 0 controls with TB diagnosed within 3 years of interview and 10 cases and 3 controls with missing data on year of TB diagnosis. The odds ratio for lung cancer associated with prior tuberculosis was 1.5 (95% CI = 1.2–1.8), after controlling for smoking, age, sex, education and occupation. The OR for males (1.4; 95% CI 1.1–1.8) and females (1.6, 95% CI 1.1–2.3) were comparable. The risk of lung cancer varied, however, according to the time when the TB was first diagnosed (Table II). The ORs were highest among those with TB diagnosed within 20 years of interview. This pattern was not simply due to the high frequency of patients over age 30 when TB was identified and treated, since the OR for lung cancer among those diagnosed with TB within the past 20 years remained high when restricting the comparison to those over age 30 at TB diagnosis. The effect of recency of TB diagnosis also persisted regardless of the method of diagnosis of lung cancer, with OR associated with TB infection in the past 20 years exceeding 2.5 regardless of whether the diagnosis of lung cancer was based on tissue examination, cytology, or radiologic/clinical grounds.

Although the OR were adjusted for smoking, there was little confounding by smoking of the TB–lung cancer association. Indeed, among controls, the smoking histories of men with and without TB were comparable; 72% of those with TB had smoked compared to 74% of those without TB, and the percentages of heavy smokers were 7% and 6%, respectively. Among female controls 18% had smoked whether or not they had TB. We also examined the consistency of the TB–cancer association across smoking categories. As shown in Table III, the ORs for lung cancer were elevated among those with a TB diagnosis in every smoking category, including lifetime non-smokers. Furthermore, the ORs associated with TB did not vary greatly across the smoking categories, with the greatest risk in each category linked to recent (within 20 year) TB infections.

**Table II** Odds ratios for lung cancer associated with prior tuberculosis infection

| | | | *Crude* | *Adjusted*[a] | |
|---|---|---|---|---|---|
| | *Cases* | *Controls* | *OR* | *OR* | *95% CI* |
| Prior diagnosis of TB | | | | | |
| No | 1105 | 1279 | 1.0 | 1.0 | – |
| Yes[b] | 266 | 213 | 1.4 | 1.5 | (1.2–1.8) |
| Years since diagnosis of TB | | | | | |
| 3–9 | 26 | 12 | 2.5 | 2.5 | (1.2–5.2) |
| 10–19 | 46 | 18 | 3.0 | 2.8 | (1.6–5.0) |
| 20–29 | 103 | 105 | 1.1 | 1.1 | (0.8–1.5) |
| 30+ | 91 | 78 | 1.4 | 1.5 | (1.0–2.1) |

[a]Adjusted for smoking, age, education, and sex; [b]Excludes 10 cases and 3 controls with missing data on year of TB diagnosis and 24 cases diagnosed within 3 years of interview.

**Table III** Odds ratios for lung cancer by years since TB diagnosis according to smoking status

| *Smoking category* | *Years since TB diagnosis* | *Cases* | *Controls* | *OR* | *95% CI* |
|---|---|---|---|---|---|
| Non-smoker | No TB | 415 | 714 | 1.0[a] | – |
| | 20+ | 43 | 83 | 1.0 | (0.7–1.5) |
| | <20 | 18 | 9 | 3.5 | (1.5–8.0) |
| Light smoker | No TB | 257 | 331 | 1.0 | – |
| | 20+ | 45 | 57 | 1.2 | (0.8–1.8) |
| | <20 | 14 | 15 | 1.5 | (0.7–3.1) |
| Moderate smoker | No TB | 310 | 197 | 1.0 | – |
| | 20+ | 85 | 32 | 1.9 | (1.2–3.1) |
| | <20 | 31 | 6 | 4.2 | (1.7–20.3) |
| Heavy smoker | No TB | 113 | 37 | 1.0 | – |
| | 20+ | 21 | 11 | 0.7 | (0.3–1.7) |
| | <20 | 9 | 0 | ∞ | — |
| Total | No TB | 1105 | 1279 | 1.0[b] | – |
| | 20+ | 194 | 183 | 1.2 | (1.0–1.6) |
| | <20 | 72 | 30 | 2.7 | (1.7–4.3) |

[a]Adjusted for age, education and sex; [b]Adjusted for smoking, age, education and sex.

TB-related risks were also examined for specific cell types of lung cancer and for peripheral *vs.* central lung cancer. As shown in Table IV, the risks associated with recent TB were increased both for adenocarcinoma and squamous/oat cell carcinoma. Significantly elevated risks were also found for both peripheral and central lung cancers. However, the risk for peripheral lung cancer was greater, with nearly two-thirds of the tumours being peripheral in patients with a recent TB infection. Furthermore, the laterality of TB and lung cancer was significantly ($P<0.01$) correlated (Table V). When TB affected the left (right) lung, the patients more often tended to have cancer on the left (right) side. The closer associations of recent TB to adenocarcinoma and to peripheral lung cancers was evident for both men and women, but the laterality effect was limited to males.

The risks associated with various drug treatments for tuberculosis are presented in Table VI. Most TB patients (82%) were treated with isoniazid (INH), but no increased risk was found for persons treated with INH alone or in combination with other medications. Few of the cases or controls with TB reported receiving artificial pneumothorax and accompanying fluoroscopy.

**Table IV** Odds ratios for lung cancer by histology, location, and years since TB diagnosis

| | *No TB* | | *Years since TB diagnosis* | | | | | |
|---|---|---|---|---|---|---|---|---|
| | | | *20+ Years* | | | *<20 Years* | | |
| | *N* | *OR* | *N* | *OR* | *95%CI* | *N* | *OR* | *95%CI* |
| Histologic type | | | | | | | | |
| Adenocarcinoma | 448 | 1.0 | 61 | 1.1 | (0.8–1.5) | 30 | 3.2 | (1.9–5.5) |
| Squamous/oat cell carcinoma | 406 | 1.0 | 94 | 1.4 | (1.0–1.9) | 30 | 2.6 | (1.5–4.6) |
| Tumor location | | | | | | | | |
| Peripheral | 493 | 1.0 | 85 | 1.2 | (0.9–1.7) | 43 | 3.8 | (2.3–6.2) |
| Central | 390 | 1.0 | 77 | 1.3 | (1.0–1.9) | 22 | 2.2 | (1.2–4.1) |

OR adjusted for smoking, age, education and sex.

**Table V** Sides of lung affected by TB and cancer

| | *TB location*[a] | | | | | | | |
|---|---|---|---|---|---|---|---|---|
| | *Left side* | | *Right side* | | *Both sides* | | *No TB* | |
| Lung cancer | No. | % | *No.* | % | *No.* | % | *No.* | % |
| Left side | 39 | 52.0 | 35 | 31.5 | 14 | 35.0 | 370 | 38.3 |
| Right side | 29 | 38.7 | 65 | 58.6 | 23 | 57.5 | 477 | 49.4 |
| Both sides | 7 | 9.3 | 11 | 9.9 | 3 | 7.5 | 119 | 12.3 |
| Total | 75 | 100.0 | 111 | 100.0 | 40 | 100.0 | 966 | 100.0 |

[a]Excludes 40 cases with missing data on location of TB or lung cancer.

**Table VI** Odds ratios for lung cancer associated with drugs used by tuberculosis patients

| *Drug* | *Cases* | *Controls* | *OR*[a] | 95%*CI* |
|---|---|---|---|---|
| INH | | | | |
| Never | 51 | 34 | 1.0[b] | – |
| Ever | 215 | 179 | 0.7 | (0.4–1.2) |
| Streptomycin | | | | |
| Never | 113 | 94 | 1.0[b] | – |
| Ever | 153 | 119 | 1.0 | (0.6–1.4) |

[a]Adjusted for smoking, age, education, sex, and years since TB diagnosis; [b]Referent category.

### Discussion

This population-based study, in an area of the world where lung cancer rates are comparatively high and where one-fifth of the male population aged 40–64 has reported tuberculosis, suggests that pulmonary TB is a risk factor for lung cancer. The TB–lung cancer relation persisted after controlling for smoking, with the risk of lung cancer in excess of 2.5-fold among individuals with a TB infection within the past two decades.

It seems unlikely that the association is simply due to the misdiagnosis of TB in persons with early-stage lung cancer, since the analysis included only TB patients diagnosed with TB 3 or more years before the lung cancer was detected. Few individuals with TB diagnosed in the 1970s or earlier could have actually had a lung tumour clinically silent until the mid 1980s, since the 5-year survival rate for lung cancer is only about 7 percent in Shanghai (Shanghai Cancer Institute, 1982). Conversely, the diagnosis of lung cancer in 1984–86 among some cases may have been incorrect, especially among persons with chronic lung diseases such as TB. We have some concern about the accuracy of the cancer diagnosis, since confirmation was based on a histologic examination of tumour tissue only for 62% of the male and 43% of the female patients. The association between lung cancer and prior TB was still seen, however, when the analysis was restricted to cases with pathologic confirmation. Furthermore, most of those without pathology reviews of tumour tissue had sputum cytology examinations, so that only a small fraction of the cases were diagnosed only on clinical and radiologic grounds.

It is possible that patients with clinically diagnosed TB, particularly in recent years, were under intense medical surveillance and thus more likely to have a lung cancer diagnosed. This type of bias could be serious if lung cancer often went undetected in the general population. Such is probably not the case, however, since lung cancer rates in Shanghai are high, not low, by world standards (Waterhouse *et al.*, 1982). In addition, we stratified the cases and controls according to number of chest X-rays during their lifetimes, one measure of medical surveillance relevant to the detection of lung cancer, and found the lung cancer–TB relation held regardless of X-ray frequency. Furthermore, the ascertainment of lung cancer in this case-control study was thought to be quite complete. All hospitals in Shanghai were checked for lung cancer admissions, not just anti-TB or chest hospitals where former TB patients would have been treated. In addition, the high response rates (99% of all living patients were interviewed) yielded a study sample nearly equivalent to the total population of all diagnosed incident lung cancer cases in Shanghai.

Another methodologic concern was the self-reporting of tuberculosis by the respondents themselves, and the possibility that cases would be more likely than the controls to recall and report TB. It seems unlikely that this type of bias is large, however, for the following reasons. Firstly, Shanghai has had an active, anti-TB program, conducting periodic mass X-ray screenings and offering standardized methods of treatment. The diagnosis of TB generally required the identification of acid-fast bacilli from bronchial secretions together with evidence from chest radiographs and physical signs. The TB patients were often isolated and given combined drug therapy, usually INH, streptomycin and para-aminosalicylic acid for 3 or more months. Thus, since TB was a relatively major life event, it is likely to be easily remembered. Secondly, we sought past medical records on 43 lung cancer patients who reported TB infections within the past 15 years. For 21, records were found in TB registers confirming the diagnosis. Among the remainder, visits to families yielded confirmation for an additional 17, but not for 2 cases, while the TB status of 3 was unknown. Hence some confirmation was provided for 38 of the 43 patients. In addition, this independent review found a nearly 90% verification of the side of the lung affected with TB.

It is also possible that the lung cancer–TB association in our study may be influenced by confounding risk factors for lung cancer. In the analysis, however, we did adjust for the effects of smoking, the dominant cause of lung cancer among men and a significant contributor among women in Shanghai. The association with TB was observed among non-smokers and several categories of smokers. Social class, as measured by education and occupation, was also taken into account. Although poor nutritional status may be a risk factor for lung cancer (Colditz *et al.*, 1987), in our study the relationship of nutrient intake to lung cancer risk was inconsistent.

The increased risks of lung cancer associated with previous TB were observed regardless of cell type and location. However, the risks associated with a recent history of TB (within 20 years of lung cancer diagnosis) were higher for adenocarcinoma than squamous/oat cell cancers, with most of the tumours arising in peripheral locations. These findings are consistent with pathologic and clinical observations linking TB lesions with lung cancer (Auerbach *et al.*, 1979; Bakris *et al.*, 1983). Furthermore, among men with both TB and lung cancer, there was a strong anatomic correlation with both lesions tending to occur on the same side of the lung. This correlation was not evident for females, but the numbers of cases were smaller and information about the localization of the tumour and TB was more often missing.

If the TB–lung cancer association is causal, the greater risk of recent infection raises the possibility that TB may act as a promoting or late-stage event in the carcinogenic process. The precise mechanisms are unclear, but perhaps the chronic TB inflammatory process potentiates the effects of other carcinogenic exposures, traps the carcinogens in scar tissue, evolves directly into a precacerous lesion, or enhances abnormal cellular proliferation and growth. On the other hand, it is also possible that the stronger link to recent TB may simply be related to competing risk and dose-response considerations. Severe TB infection in the pre-chemotherapy era was often fatal, so patients diagnosed in the 1950s and earlier would be less likely to survive to develop lung cancer. Those with TB diagnosed more than 20 years ago and still alive in the mid 1980s may have had, on average, less severe infections than those with recent diagnoses. We have used 20 years as the dividing line between recent and past TB infections not because of *a priori* hypotheses but rather because of the sharp rise in the OR for lung cancer among those with TB diagnosed within 10–19 or 3–9 compared to 20+ years of cancer diagnosis. Thus, inferences regarding the aetiologic significance of differences in risk above and below this data-derived 20-year cutoff must be interpreted cautiously.

Despite the limited number of epidemiologic studies evaluating the relationship between lung cancer and TB, most have suggested a moderate increase in the risk of lung cancer among TB patients. The largest was a cohort study of 64,000 TB patients in Canada to evaluate the late effects of INH (Howe *et al.*, 1979). A 1.5-fold excess risk of lung cancer was found in patients with TB as compared to the general population. Cohort studies in Australia (Campbell & Guilfoyle, 1970; Campbell, 1961), Denmark (Clemmesen & Jensen, 1979) and Israel (Steinitz, 1973) revealed 2-fold or greater lung cancer risks among TB patients, with the Australian study suggesting that the excess risk was not confounded by smoking. On the other hand, little evidence of an increased risk of lung cancer was detected in American (Boice & Fraumeni, 1980; Hammond *et al.*, 1967) or British (Stott *et al.*, 1976) surveys of TB patients, although the numbers of expected cases were not large. A case-control study in Hawaii revealed an 8-fold excess risk of lung cancer in nonsmoking women with a history of TB, but only 4 cases were affected (Hinds *et al.*, 1982). None of these investigations reported on the temporal association between TB and lung cancer, so that further studies are needed to evaluate our finding of enhanced cancer risk associated with recent infections. As in previous studies, we found no evidence that use of INH increases the risk of lung cancer, although the drug produces lung tumours in mice (International Agency for Research on Cancer, 1974). Since few patients had repeated chest fluoroscopies associated with artificial pneumothorax, the excess risk of lung cancer could not be attributed to this source of radiation exposure.

In summary, the data from this large population-based case-control study with exceptionally high response rates and nearly complete coverage of the Shanghai population suggest that TB is a risk factor for lung cancer, especially for TB diagnosed within the past 20 years. If the link between lung cancer and prior TB is a causal one, we estimate from calculations of attributable risk that TB would account for less than 10% of the lung cancers today in Shanghai. Thus, despite the predilection of TB patients to develop lung adenocarcinoma, it seems that the high prevalence of the infection explains only a small part of the exceptionally high rates of adenocarcinoma among nonsmoking Chinese women (Koo *et al.*, 1985). Further research is needed to clarify the role of TB in lung cancer risk and the mechanisms by which the disease may enhance carcinogenesis.

Supported in part by NCI Contract N01-CP2-1012. We thank Dr B.J. Stone, Ms Li Koo, and Ms Ruth Parsons for computer support, and Dr Brian Henderson for helpful suggestions.

### References

AUERBACH, O., GARFINKEL, L. & PARKS, V.R. (1979). Scar cancer of the lung, increase over a 21 year period. *Cancer*, **43**, 636.

BAKRIS, G.L., MULOPULOS, G.P., KORCHIK, R., EZDINLI, E., RO, J. & YOON, B. (1983). *Cancer*, **52**, 493.

BOICE, J.D. & FRAUMENI, J.F. JR. (1980). Late effects following isoniazid therapy. *Am. J. Public Health*, **70**, 987.

BRESLOW, N. & DAY, N.E. (1980). *The analysis of case-control studies*. IARC Scientific Publ. No. 32, Lyon.

CAMPBELL, A.H. (1961). The association of lung cancer and tuberculosis. *Aust. J. Med.*, **10**, 126.

CAMPBELL, A.H. & GUILFOYLE, P. (1970). Pulmonary tuberculosis, isoniazid and cancer. *Br. J. Dis. Chest*, **64**, 141.

CLEMMESEN, J. & JENSEN, S.H. (1979). Is isonicotine acid hydrazide (INH) carcinogenic to man? *Ecotoxicol. Environ. Safety*, **3**, 439.

COLDITZ, G.A., STAMPFER, M.J. & WILLET, W.C. (1987). Diet and lung cancer: A review of the epidemiologic evidence in humans. *Arch. Intern. Med.*, **147**, 157.

HAMMOND, E.C., SELIKOFF, I.J. & ROBITZEK, E. (1967). Isoniazid therapy in relation to later occurrence of cancer in adults and in infants. *Br. Med. J.*, **2**, 792.

HINDS, M.W., COHEN, H.I. & KOLONEL, L.N. (1982). Tuberculosis and lung cancer risk in nonsmoking women. *Am. Rev. Respir. Dis.*, **125**, 776.

HOWE, G.R., LINDSAY, J., COPPOCK, E. & MILLER, A.B. (1979). Isoniazid exposure in relation to cancer incidence and mortality in a cohort of tuberculosis patients. *Int. J. Epidemiol.*, **8**, 305.

KOO, L.C., HO, J.H. & LEE, N. (1985). An analysis of some risk factors for lung cancer in Hong Kong. *Int. J. Cancer*, **35**, 149.

INTERNATIONAL AGENCY FOR RESEARCH ON CANCER (1974). *Evaluation of the carcinogenic risk of chemicals to man*, Vol. **4**, IARC: Lyon.

SHANGHAI CANCER INSTITUTE (1982). Analysis of cancer incidence, survival and mortality rates in Shanghai urban area, 1972–1979. *Shanghai Tumor*, **2**, 1.

STEINITZ, R. (1965). Pulmonary tuberculosis and carcinoma of the lung. *Am. Rev. Respir. Dis.*, (Suppl) **92**, 758.

STOTT, H., PETO, J., STEPHENS, R. & 5 others (1976). An assessment of the carcinogenicity of isoniazid in patients with pulmonary tuberculosis. *Tubercle*, **57**, 1.

WATERHOUSE, J., MUIR, C., SHANMUGARATNAM, K. & POWELL, D. (eds) (1982). *Cancer Incidence in Five Continents*, Vol. **IV**, IARC: Lyon.